# An assessment of parents’ knowledge and awareness regarding paracetamol use in children: a cross-sectional study from Palestine

**DOI:** 10.1186/s12889-021-10432-5

**Published:** 2021-02-18

**Authors:** Aiman Daifallah, Ruba Jabr, Faraj Al-Tawil, Moutaz Elkourdi, Ziad Salman, Amer Koni, Ahmad Samara, Samah W. Al-Jabi, Sa’ed H. Zyoud

**Affiliations:** 1 Department of Clinical and Community Pharmacy, Department of Pharmacy, College of Medicine and Health Sciences, An-Najah National University, Nablus, 44839 Palestine; 2 Department of Medicine, College of Medicine and Health Sciences, An-Najah National University, Nablus, 44839 Palestine; 3 Poison Control and Drug Information Center (PCDIC), College of Medicine and Health Sciences, An-Najah National University, Nablus, 44839 Palestine; 4 Clinical Research Center, An-Najah National University Hospital, Nablus, 44839 Palestine

**Keywords:** Paracetamol, Acetaminophen, Knowledge, Awareness, Children, Parents, Toxicity, Palestine

## Abstract

**Background:**

Paracetamol, also known as acetaminophen, is one of the most common antipyretic and analgesic over-the-counter (OTC) medicines administered to children due to its efficacy, safety, and availability in many pharmaceutical forms, including suppositories, syrup, and drops. Parents frequently administer the wrong dose of paracetamol by mistake for their children, as reported by many previous studies. We aimed in this study to assess parents’ knowledge, attitudes, and practice regarding paracetamol dosing and toxicity, as well as their awareness regarding paracetamol-containing products.

**Methods:**

This was a cross-sectional study that targeted parents of children seeking healthcare services at primary health care centers in the Nablus area in the West Bank, Palestine. We used questionnaire-based interviews with parents for data collection.

**Results:**

A total of 300 parents were included in the study. Most of the caregivers surveyed were (87%) females (mothers). About half the parents (50.9%) reported previously using paracetamol as an antipyretic in children under the age of six. A quarter (25.4%) preferred the syrup forms, while 33.8% preferred the suppository dosage form. Medical personnel was the primary source of information for half the caregivers (51.2%). The mean knowledge score about paracetamol was 2.1 (SD = 1.4) out of 6, and the median was 2.0 with an interquartile range of 1.0–3.0. Two hundred seventy-four (95.5%) of the participants scored less than 80% and were considered to have insufficient knowledge. Only 50.9% of parents recognized that paracetamol overdose could result in serious harm.

**Conclusions:**

We found a serious lack of knowledge regarding paracetamol dosing, administration, and potential toxicity among Palestinian parents. We recommend raising awareness regarding this problem among healthcare providers and authorities and working on plans that aim to provide caregivers with accurate and adequate information on dosing, formulation, side effects, and other aspects of paracetamol use, as well as developing effective educational plans targeting healthcare providers, as well as the public.

**Supplementary Information:**

The online version contains supplementary material available at 10.1186/s12889-021-10432-5.

## Background

Paracetamol, also known as acetaminophen, is one of the most common antipyretic and analgesic over-the-counter (OTC) medicines administered to children due to its efficacy, safety, and availability in many pharmaceutical forms, including suppositories, syrup, and drops [1]. Some parents lack the necessary knowledge concerning paracetamol administration, adverse effects, dosing, and toxicity, which may lead to paracetamol under-dosing or over-dosing, which both have negative consequences on the health of children and community healthcare system outcomes [2–5]. For example, under-dosing may lead to inadequate fever management and further unnecessary consultation; making incorrect assumptions about fever causes increasing healthcare costs, resulting in unnecessary use of antibiotics [6, 7]. On the other hand, overdosing may cause dangerous side effects and toxicity, leading to hepatotoxicity and liver failure, especially in patients at risk for such injury [8–10].

As paracetamol is readily available in many brands and combinations, accidental overdose is potentially life-threatening and should be considered a significant public health concern [11–13]. Rising rates of intentional and accidental paracetamol poisoning in childhood [14, 15] make it imperative that the public be aware of paracetamol overdose risks. Parents frequently administer the wrong dose of paracetamol by mistake for their children, as reported by many previous studies. Many contributing factors for this problem have been suggested, including the widespread use of paracetamol, its availability in many forms, low levels of awareness regarding the active components in paracetamol products, and misconceptions about the potential of OTC medications for harm [3, 4, 16–23]. Additionally, many studies also noted that parents have inadequate knowledge of paracetamol use and poor awareness of its toxicity [3–5, 16–25].

Due to these important public health implications, parents’ knowledge about paracetamol is important as paracetamol is considered the most encountered individual pain medication used (42.2% of all pain medications used) in Palestine [15, 26–28]. Furthermore, paracetamol poisoning was the most frequently reported case by emergency departments in Palestine [29]. Additionally, paracetamol toxicity lies at the top of the medication poisoning list in many countries [30, 31]. A US study from 2011 found that both intentional and accidental poisoning of paracetamol continues to be a major public health problem [32]. Parents may seek information about paracetamol from health care professionals (doctors or pharmacists), medication package inserts and product labels, advertisements, the internet, family and friends, and drug call centers [33, 34].

A previous study conducted in Palestine showed that medications, mainly pain medications, are responsible for accidental poisoning and suicidal cases [26]. Indeed, this study analysed the calls received by the Palestinian Poison Control and Drug Information Center (PCDIC) over 2 years and revealed that unintentional poisoning was the leading reason for the calls (62.8%) and, importantly, the majority of victims (54.0%) were under the age of 6 years [32]. Another analysis of a medical call center conducted in Australia found that consumers have many questions and concerns regarding paracetamol safety and efficacy underestimated by healthcare professionals [34]. A study that evaluated Palestinian parents’ knowledge, attitudes and practice regarding self-medication for their children found that 67.4% of responders believed that paracetamol is safe, even in very high doses [35]. This misconception is very dangerous because high doses of paracetamol can lead to liver damage.

In Palestine, many studies have assessed medication use in children [35–39], but none addressed parents’ awareness and knowledge regarding paracetamol dosage forms. Therefore, in the current study, we aimed to assess parents’ knowledge, attitudes, and practice regarding paracetamol dosing and toxicity, as well as their awareness regarding paracetamol-containing products. Since accidental ingestions have been linked with a greater risk of hepatotoxicity, awareness regarding paracetamol-containing products is of public health importance. Our study results may be used as a basis for a more extensive survey, which can help responsible authorities implement regular educational programs and make appropriate interventions according to the study results. Moreover, this study’s results offer vital data for local health-related policy-makers and educational institutions in their efforts to achieve evidence-based and safe medical practices by increasing parents’ awareness and knowledge on the topic of this study.

## Methods

### Study design and setting

A cross-sectional study was conducted in primary healthcare centers in the Nablus area (including Nablus city and nearby refugee camps and villages). We collected data from pediatrics’ health care centers, where parents went to vaccinate their children regularly and then focused on dates when we could hire the needed sample. Most of the cases that come to these clinics do not have serious symptoms. Clearly, cases with serious symptoms mainly visit the emergency department. Data was collected over a 3-week from December 2016 in mid-winter. Four primary health care centers were selected for this study. These centres have the following in common, making them ideal as a field of research [37–40]: (1) provide a complete primary health care package; (2) serve a wide number of patients; and (3) cover three types of populations in the area (rural, urban, and Palestinian refugee camps).

### Sample size and sampling technique

The total population of the Nablus area was 340,117, with 187,839 living in Nablus city. During the study period, the approximate number of parents visiting the pediatrics’ health care centers for vaccination was 1200. This number was used as a population to decide the sample size needed for analysis. The sample was calculated by the Daniel formula [41], *n =* Z^2^*P (1-P) / d^2^, where *n =* sample to be calculated, z = 1.96 (CI of 95%), d = 0.05 (absolute precision as a margin of error), *P* = 0.5 expected prevalence or response distribution. Thus, the calculated sample size was around 385 subjects. This number was used as the sample size when the population of the study is more than 10,000. In our study the sample size needs to be adjusted as our population is relatively small, *N* = 1200 (*N* < 10,000). Therefore, the required minimum sample will be obtained by using the following equation; adjusted sample = n/ (1 + (n/N)), where n: is the sample size when the population is more than 10,000 and N is the population. The adjusted sample was around 292 subjects. In addition, to ensure adequate power and enhance study reliability, the target sample size was increased to 300 participants. We selected all primary healthcare centers which are distributed in the study area covering all geographical clusters to obtain a sample with adequate representation. The goal of the sampling procedure was to have an equal sample size from each primary health center, in which a sample of 75 parents was collected using a convenience sampling technique from each primary health center.

### Inclusion and exclusion criteria

In the current study, we included parents between the ages of 19 and 48 years who parented one or more children aged 6 years or younger. Subjects were also required to read and agree to the study’s informed consent. In some cases where the participants were with no formal education, we provided them with verbal informed consent with a complete explanation of all study aspects. After parents agreed to participate, an appropriate time and comfortable place for the interview was selected. We excluded subjects whose children had one or more chronic illnesses, such as cancer, congenital heart disease, cerebrovascular disease, psychiatric disorders, and hepatic diseases. Subjects whose children were hospitalized before for at least 5 days and were also excluded. In addition to the exclusion of pediatric emergency sites in hospitals.

### Data collection instrument

Parents were interviewed using a questionnaire (Additional file 1) that was developed by retrieving and defining essential domains from the relevant literature to assess parents’ knowledge, attitudes, and practice regarding paracetamol dosing and toxicity, as well as their awareness regarding paracetamol-containing products [3–5, 16–25, 37]. The interview was achieved by four pharmacists who had experience in community pharmacy practice and counseling with patients. These specialists were chosen to guarantee the language’s simplicity and make the applied instrument more clear and understandable to the parents. Clearly, the investigators explained each question to the subjects throughout the interview. Moreover, to ensure the questionnaire’s content validity and to confirm that the items can be understood, it was reviewed and validated by three experts in pharmacy practice. To check for any possible improvements in the questionnaire, a pilot study was performed with 15 parents, but as the chosen parents all understood the questionnaire, no adjustments were made. Initially, and before providing the participants with the questionnaire, we asked them, “Which one of you primarily provided health care to the child” and accordingly, we interviewed one parent (father or mother) in case both parents were existing.

The questionnaire contained socio-demographic items (e.g., age, gender, level of education for parents, and the number of children in the family). Initially, the parents were asked if they used paracetamol previously. Furthermore, they were shown photos of the outer box of certain products, some of which contained paracetamol, to point out the products which contain paracetamol. The purpose of these two steps was to ensure the parents know paracetamol medication. In addition, parents were asked about items regarding paracetamol dosage forms (suppositories, syrups, and drops) that were available in Palestine to determine the doses used and caused beyond their choice (i.e., ease of administration of the suppository dosage form). Moreover, all trade names of paracetamol available in Palestine were used in the study to avoid bias based on the variable use of these products. Finally, subjects’ knowledge of paracetamol toxicity was measured by a set of multiple-choice items that covered the following topics: recommendations for a daily dose, specific side effects, and overall potential to cause harm. Importantly, we provided the parents with an educational orientation at the end of the interview for incorrect answers that may harm their children. A knowledge score was calculated as the sum of correct answers to items that covered paracetamol administration, dosing, and toxicity and ranged from 0 to 6, with higher scores representing better knowledge.

### Statistical analysis

Data were analyzed using the Statistical Package for Social Sciences (SPSS), version 21. We conducted a descriptive analysis of characteristics and question items. Frequencies and percentages were calculated for categorical variables, and means and standard deviations and/or medians and interquartile were calculated for numerical variables. Based on Bloom’s cut off point, parents’ overall knowledge was categorized as sufficient knowledge if the score was above 80% (≥5 points) and poor if the score was less than 8% (< 5 points) [42].

## Results

### Socio-demographic data of parents and their children

Three-hundred parents of children aged 6 years or younger participated in this study. Most participants (87%) were female parents and three quarters (75.3%) of mothers’ age were between 19 and 30 years old. More than half of the mothers (55.3%) had completed high school education as their highest level of education. A third of the participants (33.0%) had two children below 18 years of age, and a half (50.0%) had two children below 6 years of age. Of the children presenting to their parents, 54% were boys, and 43.7% had one doctor visit in the last 6 months. The participating parents and their children’s demographic features are presented in Tables 1 and 2, respectively.

**Table 1:** Socio-demographic characteristics of children

Variable ^**a**^	Frequency (%)
**Gender**	
Male	162 (54.0)
Female	138 (46.0)
**Child order**	
First	98 (32.7)
Second	107 (35.7)
Third	56 (18.7)
Fourth	19 (6.3)
**Number of doctor’s visits in the last 6 months**	
None	10 (3.3)
Once	131 (43.7)
Twice	61 (20.3)
Three times	53 (17.7)
Four times or more	35 (11.7)

^a^There were missing data on some of the variables

**Table 2:** Socio-demographic characteristics of parents

Variable ^**a**^	Frequency (%), ***N*** = 300
**Number of children aged less than six years**	
1 child	115 (38.3)
2 children	150 (50.0)
3 children or more	35 (11.6)
**Number of children aged < 18 years**	
None	67 (22.3)
1 child	76 (25.3)
2 children	99 (33.0)
3 children	29 (9.7)
4 children or more	28 (9.3)
**Fathers’ age (year)**	
21–30	126 (42)
31–40	137 (45.7)
41–56	35 (11.7)
Dead	2 (0.7)
**Mothers’ age (year)**	
19–30	226 (75.3)
31–40	64 (21.3)
41–56	10 (3.3)
**Relationship**	
Fathers	37 (12.3)
Mothers	261 (87)
**Father’s educational level**	
Uneducated	13 (4.3)
Elementary school (primary)	26 (8.7)
Middle school (junior high school)	110 (36.7)
High school (secondary school)	142 (47.3)
University	9 (3.0)
**Mother’s educational level**	
Uneducated	4 (1.3)
Elementary school (primary)	11 (3.7)
Middle school (junior high school)	114 (38.0)
High school (secondary school)	166 (55.3)
University	5 (1.7)

^a^There were missing data on some of the variables

### Awareness regarding paracetamol products and indications

Half the participants (50.7%) reported previous paracetamol administration to their children without consulting a doctor (Table 3). Of the 287 participating parents who previously used paracetamol or could identify at least one paracetamol product, 50.9% reported that they used paracetamol to reduce fever, and 15.3% used it as an analgesic (Table 4). Most participants reported that using paracetamol without a prescription was the experience of the child’s previous illness.

**Table 3:** Awareness regarding paracetamol products among the participants

Variable	Frequency (%); ***N =*** 300
**Used paracetamol for the child without consulting a doctor**	
Yes	152 (50.7)
No	75 (25.0)
I do not know what paracetamol is	73 (24.3)
**Used one of the following products for the child**	
Acamoli cold	104 (34.6)
Acamoli syrup 250 mg/5 ml	101 (33.7)
Acamoli syrup 125 mg/5 ml	139 (46.3)
Acamoli supp 80 mg	113 (37.7)
Acamoli supp 150 mg	118 (39.3)
Acamoli forte supp 250 mg	69 (23.0)
Paramol infant supp	64 (21.3)
Paramol extra susp	79 (26.3)
Paramol child supp	112 (37.3)
Paramol plus	30 (10.0)
Paramol extra	28 (9.3)
Otamol syrup	78 (26.0)
Otamol supp	68 (22.7)
Flu syrup	104 (34.7)
Novimol drops	46 (15.3)
Sedamol tab	48 (16.0)
Panadol tab	64 (21.3)
Dexamol tab	31 (10.3)
Flu tab	35 (11.6)
**Which product(s) contain(s) paracetamol**	
Acamoli cold (available)	61 (20.3)
Acamoli syrup 250 mg/5 ml (available)	104 (34.7)
Acamoli syrup 125 mg/5 ml (available)	136 (45.3)
Acamoli supp 80 mg (available)	90 (30.0)
Acamoli supp 150 mg (available)	107 (35.7)
Acamoli forte supp 250 mg (available)	93 (31.0)
Paramol infant supp (available)	76 (25.3)
Paramol extra susp (available)	81 (27.0)
Paramol child supp (available)	106 (35.3)
Paramol plus (available)	67 (22.3)
Paramol extra tab (available)	71 (23.7)
Otamol syrup (available)	88 (29.3)
Otamol infant supp (available)	80 (26.7)
Flu syrup (not available)	41 (13.7)
Flu tab (available)	36 (12.0)
Novimol drops (available)	46 (15.3)
Trufen (not available)	60 (20.0)
Adcef (not available)	30 (10.0)
Amoxitid (not available)	35 (11.6)
Dexamol (available)	44 (14.7)
Sedamol (available)	46 (15.3)
Acamol tab (available)	91 (30.3)
Panadol tab (available)	96 (32.0)
Moxepharm (not available)	40 (13.3)
Emegrain (available)	32 (10.7)

**Table 4:** Paracetamol indications as perceived by the participating parents

Paracetamol use	Frequency (%); ***N*** = 287
Anti-pyretic	146 (50.9)
Analgesic	44 (15.3)
Sedative	15 (5.2)
Symptoms of illness (cough, flu, and vomiting)	18 (6.3)
Anti-pyretic and analgesic	25 (8.7)
Anti-pyretic and sedative	2 (0.7)
All the above mentioned reasons	37 (12.9)

### Participating parents’ knowledge regarding paracetamol use

The mean knowledge score was 2.1 (SD = 1.4) out of 6, and the median was 2.0, with an interquartile range of 1.0–3.0. When asked about the maximum daily number of paracetamol administration, 42.2% of the participants answered with 3, 27.2% answered with 4, 19.2% answered with 2, and 4.5% answered that it should not exceed five doses. Most (61.0%) participants selected ‘4–6 h’ as the time to be allowed between doses. Two hundred seventy-four (95.5%) of the participants scored less than 80% and were considered to have insufficient knowledge. Approximately 46% of the subjects acknowledged that paracetamol overdose could damage renal failure, liver failure, gastric irritability, and immune suppression. On the time limit after which the syrup becomes invalid, 44.6% considered this limit to be the expiry date, whereas 20.6% considered this limit to be up to 6 months after opening. Regarding the participants’ knowledge score, 9.8% of the subjects achieved a score of 0, 32.8% achieved a score of 1, whereas only 18.4% achieved a four or higher score (Table 5).

**Table 5:** Participating parents’ responses to questions regarding knowledge of paracetamol use

Variable	Frequency (%); ***N*** = 287
**Temperature at which paracetamol is given as antipyretic**	
38 ° C	111 (38.7)
38.5 ° C	77 (26.8)
39 ° C	69 (24.0)
39.5 ° C	9 (3.1)
40 ° C	1 (0.3)
I don’t know	20 (7.0)
**Daily maximum frequency of paracetamol use**	
One dose	9 (3.1)
Two doses	55 (19.2)
Three doses	121 (42.2)
Four doses	78 (27.2)
Five doses	13 (4.5)
I don’t know	11 (3.8)
**Time allowed between doses**	
Less than 4 h	18 (6.3)
4–6 h	175 (61.0)
More than 6 h	78 (27.2)
I don’t know	16 (5.6)
**Time allowed for use after opening a syrup**	
Up to 3 months	62 (21.6)
Up to 6 months	59 (20.6)
Until the expiry date	128 (44.6)
I don’t know	38 (13.2)
**Paracetamol overdose can cause serious adverse effects**	
Yes	146 (50.9)
No	66 (23.0)
I don’t know	75 (26.1)
**Type of damage paracetamol overdose causes**^**a**^	
Renal failure	46 (31.5)
Liver damage	42 (28.8)
Gastric irritability	23 (15.8)
Immune suppression	21 (14.4)
Other	14 (9.5)
**Knowledge Score (out of 6)**	
0	28 (9.8)
1	94 (32.8)
2	60 (20.9)
3	52 (18.1)
4	40 (13.9)
5	9 (3.1)
6	4 (1.4)

^a^Only participants who agreed with the previous item answered this one

### Parents’ paracetamol-related practices

Table 6 summarizes the participating parents’ answers on items that assessed paracetamol-related practices. A third (33.8%) of the subjects preferred suppository dosage forms, a fourth (25.4%) chose syrup as their preferred dosage form, and only 1.7% used paracetamol drops for their children. Furthermore, this study reported that some parents have difficulties in administering medications to their children. Child refusal to swallow the medication was the difficulty most encountered by parents during paracetamol administration to children. The majority of the participating parents (51.2%) reported that health care providers (doctors and pharmacists) were their primary source of information on medication use.

**Table 6:** Participating parents’ responses to questions on paracetamol-related practices

Variable	Frequency (%); ***N =*** 287
**Pharmaceutical dosage form preferred**	
Syrup	73 (25.4)
Suppositories	97 (33.8)
Syrup and suppositories	112 (39.0)
Drops	5 (1.7)
**Syrup dose used before**	
125 mg/5 ml	106 (36.9)
250 mg/5 ml	93 (32.4)
Didn’t use before	88 (30.7)
**Syrup measuring tool used before**	
Teaspoon	10 (3.5)
Table spoon	14 (4.9)
Syringe	178 (62.0)
Measuring cup	2 (0.7)
Didn’t use before	83 (28.9)
**Suppository dose used before**	
80 mg	27 (9.4)
150 mg	83 (28.9)
250 mg	48 (16.7)
300 mg	64 (22.3)
80 mg and 150 mg	6 (2.1)
Didn’t use before	59 (20.6)
**Reasons for choosing this dosage form**	
Price	11 (3.8)
Efficacy	94 (32.8)
Doctor’s recommendation	72 (25.1)
Pharmacist’s recommendation	36 (12.5)
Other’s recommendation	12 (4.2)
Easily used	35 (12.2)
Other	27 (9.4)
**Fever management if no improvement within 2 h**	
Give another dose of paracetamol	61 (21.3)
Use other medications	37 (12.9)
Use cold sponges	87 (30.3)
Consult a doctor	90 (31.4)
Use cold sponges and consult a doctor	4 (1.4)
Other	8 (2.8)
**Reasons for using paracetamol without prescription**	
I do not trust the healthcare system	10 (3.5)
No need to visit the doctor	84 (29.3)
Doctor fee is too expensive	38 (13.2)
Prior experience with similar symptoms	104 (36.2)
Other	51 (17.8)
**Difficulty with administration**	
Children refuse to swallow the medication	104 (36.2)
Child is not cooperative due to illness	42 (14.6)
Child is sleeping at the dose time	28 (9.8)
No difficulty	100 (34.8)
Other	13 (4.5)
**Ways to ensure the child has taken the medication**	
Using force	54 (18.8)
Coaxing and encouraging the child	76 (26.5)
Mixing medicine with food or drinks	48 (16.7)
Seeking medical advice	18 (6.3)
Using non-pharmacological methods	10 (3.5)
Using suppositories instead of syrup	69 (24.0)
Other	9 (3.1)
Mixing medicine with food or drinks and using suppositories	3 (1.0)
**Reason for repeating the dose**	
Severity of illness	106 (36.9)
Age	43 (15.0)
Weight	37 (12.9)
Medication leaflet	47 (16.4)
Doctor’s consultation	43 (15.0)
Pharmacist’s consultation	11 (3.8)
**Sources of information on paracetamol dose**	
Doctor’s consultation	96 (33.4)
Pharmacist’s consultation	51 (17.8)
Own knowledge	43 (15.0)
Relatives and friends	42 (14.6)
Experience from previous use	41 (14.3)
Medication leaflet	14 (4.9)
**Dose quantity is determined by**	
Weight	78 (27.2)
Age	73 (25.4)
Severity of illness	61 (21.3)
Experience from previous use	19 (6.6)
Medication leaflet and weight	52 (18.1)
Other	4 (1.4)

## Discussion

This study assessed parents’ knowledge and their choice of paracetamol dosage forms for their children. The current study drove us to highlight the most commonly used OTC drug (paracetamol) and the most age categories at risk for intoxication with paracetamol unintentionally by evaluating the knowledge of parents using paracetamol for their children. In addition, the present study was designed to have an obvious image of parents’ practices and attitudes toward paracetamol and to assess the basic knowledge of the parents. The availability of paracetamol and other analgesic medications such as OTC suggests that the bulk of the communities with mild to moderate pain symptoms are self-medicated. However, the general public needs to be aware of the potential risks of paracetamol misuse and overdose [43]. Observational studies are a perfect occasion for providing correct knowledge and inspire learning to parents and the community [44]. Therefore, health researchers can make a difference if taking advantage for health education.

We found that half of the participants (50.9%) had previously used paracetamol as an antipyretic to their children. A similar percentage (50.7%) of them reported administering paracetamol to their children without consulting a doctor. Despite these high paracetamol use rates, we found incorrect answers in some aspects regarding paracetamol administration, dose, and toxicity.

A third of the participants reported a preference for the suppository dosage form used. Ninety-four parents (32.8%) justified their choices (either syrup, suppositories, or drops) as its higher efficacy compared to other dosage forms, and a small percentage (12.2%) reported convenience as the reason behind their choice. However, the official recommendation states that the oral route for paracetamol administration is generally preferred in children, while the liquid form allows for more precision in estimating the right dose. In comparison, the rectal route results in slower and incomplete absorption [45]. Besides, rectal paracetamol products have many drawbacks, such as slower and more erratic absorption, varying blood concentration, insufficient therapeutic outcome, and a higher risk of toxicity [17].

According to our results, most parents (62%) stated that they used a syringe when measuring the dose of paracetamol syrup, whereas 8.4% admitted to the use of inaccurate measuring tools, such as tablespoons and teaspoons. This matches the findings observed in earlier studies [46], which reported that regular kitchen spoons were still being used to administer medications to children at home. This, in turn, may be contributing to the high rates of inaccurate dosing of paracetamol in young children by parents. Medical personnel was the main source of medication information among parents in our sample. This result is in concordance with the results of a similar Israeli study [17] wherein 57.2% identified medical personnel as their primary source of information on paracetamol use. On the other hand, 14.6% of parents reported that they obtained information on paracetamol from relatives and friends. Therefore, enhancing access to reliable and precise information about paracetamol is mandatory to promote the safe and effective use of the most worldwide consumed analgesics and antipyretics. Physicians, community pharmacists, and information provided by medication package inserts must provide accurate information and precise instructions to parents regarding paracetamol use. Health education and promotion campaigns should also replace other unreliable sources, such as relatives and friends, with knowledge related to appropriate paracetamol use. Most patients in Palestine seek self-medication directly from community pharmacies for mild to moderate pain because they are easier to access and less costly than physicians’ clinics. Paracetamol has become the medication choice for many patients, community pharmacies, and physicians in the treatment of mild to moderate pain and fever with its safe stomach profile, low cost, and OTC convenience [43]. Unfortunately, paracetamol’s wide variety of brands, OTC status, lack of national paracetamol poisoning data, and lack of public health education programs to raise awareness and knowledge on paracetamol in the general population have perpetuated the illusion that paracetamol is fully benign [35, 37, 43].

On the question of paracetamol overdose damage, only 50.9% of the parents in our sample were aware of potential harm from paracetamol overdose. On a related note, 21.3% of parents stated that they would give another dose of paracetamol within 2 h after the first dose if there was no improvement. This further supports the idea that dose miscalculation and lack of knowledge of the appropriate dose timing could be behind many reported cases of severe hepatic damage or even death from paracetamol products. Knowing the maximum daily dose is of great importance since most toxicity cases occur from exceeding the recommended daily dose. In this study, the majority of the parents demonstrated inaccurate knowledge in this area. These results agreed with those observed in a survey conducted in the United States in which only a third of the subjects knew the correct maximum daily dose [45]. The American Academy of Pediatrics’ recommendation states that clear written information on paracetamol should be given to caregivers during the well-child visit on multiple occasions [47].

Another interesting finding was that 36.9% of the participating parents stated that the severity of the illness was the determinant of the dose they would give. This is in collision with the recommendation regarding age-based dose scheduling, which suggests allowing for an incremental increase in the dose with growth, not with illness severity [48].

Knowledge among participants regarding paracetamol administration, dosing, and toxicity was rated by a score of 0–6, with 81.6% of our participants achieving ≤3 points. This result indicates insufficient knowledge regarding paracetamol use among caregivers. Combined with the finding that more than a third of the participants stated that they had used paracetamol without consulting a doctor because they reckoned they had had enough knowledge from experience with previous illness in children. These findings are concerning and warrant immediate interventions that aim to increase the level of awareness and provide accurate and adequate knowledge for parents and the general population regarding paracetamol use.

### Strengths and limitations

This was the first study in Palestine to assess parents’ knowledge regarding paracetamol. The study covered the topic of paracetamol use in children from different angles, including product identification, dose-related knowledge, dosage form, storage, administration, and toxicity. On the other hand, this study had some limitations. First, we used a convenient sampling for participants’ selection. Moreover, most of the study subjects were females, which may have limited the representability of our sample and they were not reviewed after the doctor’s instructions. It also limited our ability to examine any gender-related differences in parental knowledge. Because we studied only one area, our findings may not accurately reflect all parents’ situation in Palestine. Furthermore, the cross-sectional design that was adopted in the current study did not allow for testing causality between variables. In addition, the absence of control groups (i.e., parents with children versus parents without children) limits the interpretation of paracetamol burden on parents’ knowledge and awareness. Finally, the recall bias and language level of complexity could not be eliminated completely due to limitations in such studies.

## Conclusions

This study showed that there were inadequate information and awareness among Palestinian parents about the use of paracetamol. This knowledge gap was evident in different areas, including paracetamol dosing, administration timing, and toxicity potential. Accordingly, we recommend raising awareness regarding this problem among parents and working on plans to provide caregivers with accurate and adequate information on dosing, formulation, side effects, and other aspects of paracetamol use. Moreover, we recommend assessing knowledge among health care professionals regarding paracetamol as doctors and pharmacists are the main sources of information for the majority of parents. Therefore, we can ensure parents are provided with accurate and adequate information regarding medication. However, the findings shed light on parents’ behavior, strengthening their knowledge that can be used to raise public awareness about the risks of paracetamol. In addition, appropriate strategies are also needed to reduce parental errors in paracetamol administration for children. To bridge the gap in the rational use of paracetamol, healthcare providers and public health officials should work together.

## Supplementary Information

**Additional file 1.** Study questionnaires. This is the final English version of the questionnaire used to obtain data that helps to evaluate parents’ knowledge, attitudes, and practice regarding paracetamol dosing and toxicity and their awareness regarding paracetamol-containing products in Palestine.

## Data Availability

The datasets used and/or analyzed during the current study are available from the corresponding author on reasonable request.

## Abbreviations

OCT: Over the counter
IRB: Institutional Review Board
