# Supplementary material for: An assessment of parents’ knowledge and awareness regarding paracetamol use in children: a cross-sectional study from Palestine
Source: BMC Public Health. 2021 Feb 18;21:380. doi: 10.1186/s12889-021-10432-5 (PMC7890973; doi:10.1186/s12889-021-10432-5)
Supplement: Supplementary file 1 — Additional file 1. Study questionnaires. This is the final English version of the questionnaire used to obtain data that helps to evaluate parents’ knowledge, attitudes, and practice regarding paracetamol dosing and toxicity and their awareness regarding paracetamol-containing products in Palestine. [file 12889_2021_10432_MOESM1_ESM.doc]

**Additional file 1: Study questionnaires.** This is the final English version of the questionnaire that was used to obtain data that helps to evaluate parents’ knowledge, attitude, and practice regarding paracetamol dosing and toxicity, as well as their awareness regarding paracetamol-containing products in Palestine.

**Place of data collection: □ Governmental Clinic □ Private Clinic**

**First section:** Demographic data.
**=================================================================================================================================================**

**A. The Child:**

**Gender:** □ Male □ Female

**Age:** ____

**Order:** ____

**Weight: ____

Is there any hospital admission history?** □ Yes □ No

**Number of children:**

1. Less than 6 years: ____
2. Less than 18 years: ____

**Age:**
 1. 1^st^ Child: ____

2. 2^nd^ Child: ____

3. 3^rd^ Child: ____

4. 4^th^ Child: ____

5. 5^th^ Child: ____

**B. Parents:**

**Relationship:** □ Father □ Mother

**Age:** Father: **____** Mother: **____**

**Occupation:** Father: **____** Mother: **____**

**Educational level:**

Father:

□ Uneducated □ Elementary □ Middle school □ High school □ University

Mother:

□ Uneducated □ Elementary □ Middle school □ High school □ University

**Residency:** □ City □ Village □ Palestinian refugee camp

**Income level of the family:**

□Low (less than 2000 NIS) □ Average (2000-5000 NIS)

□ High (5000-10000 NIS) □ Very High (>10000 NIS)

**Health insurance type: □** Government □ Private □ None

**How many times did you take your child to the doctor in the last 6 months? _____**

**=================================================================================================================================================Second section:** Awareness regarding paracetamol products among the participants
**=================================================================================================================================================**

**Do you ever used Paracetamol for your child without consulting a physician?**

□ Yes

□ No

□ I don’t know what paracetamol is

**For the table below:**

1. **Check A if you have ever used the medication for your child.**

**B. Check B if the product contains Paracetamol.**

| **Trade name** | **A** | **B** |
| --- | --- | --- |
| Acamoli Cold | **□** | **□** |
| Acamoli 125mg/5ml | **□** | **□** |
| Paramol Extra sus. | **□** | **□** |
| Otamol syrup | **□** | **□** |
| Acamoli 250mg/5ml | **□** | **□** |
| Paramol Child sup. | **□** | **□** |
| Acamoli 150mg supp. | **□** | **□** |
| Acamoli Forte 250 mg supp. | **□** | **□** |
| Acamoli Baby 80mg supp. | **□** | **□** |
| Paramol Infant supp. | **□** | **□** |
| Otamol Inf. sup. | **□** | **□** |
| Panadol | **□** | **□** |
| Acamol | **□** | **□** |
| Tiptipot Novimol drops | **□** | **□** |
| Paramol Extra tab. | **□** | **□** |
| Paramol Plus | **□** | **□** |
| Emegrain | **□** | **□** |
| ADCEF | **□** | **□** |
| Amoxitid 750 | **□** | **□** |
| Moxepharm 400 | **□** | **□** |
| Dexamol | **□** | **□** |
| Sedamol | **□** | **□** |
| Flu tab | **□** | **□** |
| Flu syrup | **□** | **□** |
| Trufen | **□** | **□** |

**If answered “Yes” for any of the previous two questions, what is the reason for use?**

□ Anti-pyretic

□ Analgesic

□ Sedative

□ Symptoms of illness (cough, flu, and vomiting)

**□** The child doesn’t eat or drink

□ All the above mentioned reasons

**If answered “No” for any of the previous two questions, what is the reason for NOT use?**

□ No need for the drug

□ Doesn’t want to use drugs

□ The child refuse to take the drug

□ Don’t take drugs without prescription

□ Other: ______

**=================================================================================================================================================Third section:** Parents’ knowledge of paracetamol use
**=================================================================================================================================================**

**At which temperature Paracetamol is given as antipyretic without consulting a physician?**

□ 38

□ 38.5

□ 39.5

□ 40

□ Don’t know

**What’s the maximum daily number of doses of Paracetamol allowed to be given for children?**

□ 1

□ 2

□ 3

□ 4

□ 5

□ Don’t know

**How much time allowed between Paracetamol doses?**

□ Less than 4 hours

□ 4-6 hours

□ More than 6 hours

□ Don’t know

**How long can we use Paracetamol syrup after opening the bottle?**

□ Up to 3 months after opening the bottle

□ Up to 6 months after opening the bottle

□ Until the expiry date of the product

□ Don’t know

**Does Paracetamol overdose cause any harm?**

□ Yes

□ No

□ Don’t know

**If you answer “Yes” for the previous question, what would Paracetamol overdose cause?**

□ Renal failure

□ Liver damage

□ Stomach problems

□ Immunosuppression

□ Other: _______

**The dose quantity is determined by:**

□ Weight

□ Age

□ Severity of illness

□ Experience from previous use

□ Medication leaflet & Weight

□ Other

**=================================================================================================================================================Forth section:** Parents’ Paracetamol-related practices & attitudes
**=================================================================================================================================================**

**What dosage form do you prefer to use for your children?**

□ Syrup

□ Suppositories

□ Syrup & Suppositories

□ Drops

**If the answer is “Syrup” in the previous question, what is the drug strength used?**

□ 125 mg / 5 ml

□ 250 mg / 5 ml

**What is the tool you used to measure the dose?**

□ Teaspoon

□ Tablespoon

□ Syringe

□ Measuring cup

□ Other: _______

- (Point to the measuring range: _________)

**If the answer is “Suppositories”, what is the drug strength used?**

□ 80 mg

□ 150 mg

□ 250 mg

□ 300 mg

□ 80 mg & 150 mg

**What is the reason for choosing this dosage form?**

□ Price

□ Efficacy

□ Recommendation of physician

□ Recommendation of pharmacist

□ Recommendation from others

□ Easily used

□ Other

**What is the reason for using Paracetamol without a prescription?**

□ Don’t trust health care system

□ No need to visit the doctor

□ Doctors fee is too expensive

□ Prior experience with similar symptoms

□ Other

**What difficulties encountered with Paracetamol administration to the child?**

□ Child refuse to swallow the medication

□ Child is not cooperative due to illness

□ Child is sleeping at the dose time

□ No difficulties

□ Other

**What way used to ensure the child has taken the medication?**

□ Using force

□ Coaxing and encouraging the child

□ Mixing medicine with food or drinks

□ Seeking medical advice

□ Using non-pharmacological methods

□ Using suppositories instead of syrup

□ Other

□ Mixing medicine with food or drinks and using suppositories

**When repeating Paracetamol doses more than once, you depend on:**

□ Severity of illness

□ Age

□ Weight

□ Medication leaflet

□ Doctor’s consultation

□ Pharmacist’s consultation

**What sources of information about Paracetamol dose you depend on?**

□ Doctor’s consultation

□ Pharmacist’s consultation

□ Own knowledge

□ Relatives and friends

□ Experience from previous use

□ Medication leaflet

□ Other
